# Survey of Screw-Retained versus Cement-Retained Implant Restorations in Saudi Arabia

**DOI:** 10.1155/2017/5478371

**Published:** 2017-10-30

**Authors:** Alaa Makke, Abdulwahed Homsi, Montaha Guzaiz, Abdulrahman Almalki

**Affiliations:** Oral and Maxillofacial Department, Faculty of Dentistry, Umm Al-Qura University, Mecca, Saudi Arabia

## Abstract

**Introduction:**

Implant-supported prostheses are currently the standard treatment for the replacement of missing teeth and deficiencies. Implant restorations can either be screw-retained, cement-retained, or both. The implant retention system type is typically chosen during the treatment plan. The primary purpose of this study is to investigate the frequency of implant restoration retention systems.

**Materials and Methods:**

A five-page questionnaire was sent to private institutes, educational institutes, and governmental hospitals that provide dental services. The data were analyzed using descriptive statistics.

**Results:**

Prior to distribution, the surveys were proofread and pilot-tested at the Faculty of Dentistry at Umm Al-Qura University. The surveys were mailed to three groups: private institutes, educational institutes, and governmental hospitals. In total, 120 surveys were distributed and 87 surveys were returned, for a response rate of 73%. This included thirty-six surveys (41.4%) from private institutes, twenty-two surveys (25.3%) from educational institutes, and twenty-nine surveys (33.3%) from governmental hospitals.

**Conclusions:**

In general, Astra was cited as the most widely used implant system. In addition, cement-retained restorations were more frequently used than screw-retained restorations. However, dental implant failure was more frequently associated with cement-retained restorations than with screw-retained restorations.

## 1. Introduction

Implant-supported prostheses are currently the standard treatment for the replacement of missing teeth and deficiencies to enhance tooth function, for convenience, and for appearance [1]. An implant fixed prosthetic part can be screwed and/or cemented to the dental implant. The implant retention system type is typically chosen during the dental treatment plan, when the advantages and disadvantages of each system are considered [2]. In this context, patient preference may influence the retention system choice [3].

Screw-retained systems are preferred for a prosthesis with multiple abutments, due to the retrievability that allows for the removal of the prosthesis for cleaning and repair. In addition, screw-retained prosthesis tends to have less marginal misfits at the crown implant interface [4, 5]. However, the screw-retained system shows higher rates of complications (e.g., screw loosening, fracturing, and esthetic considerations) when the implants are improperly positioned [6].

Cement-retained systems are ideal for esthetic purposes. They may provide an advantage in compensating for the unfavorable angulation of an implant. Other advantages include fabrication simplicity, a decrease in laboratory complications, and less stress on bone tissue compared to the screw-retained systems [7, 8]. However, the cement-retained systems are sensitive and need more care to avoid excess cement, which can lead to surrounding soft tissue inflammation [9].

As there is currently no consensus about the ideal type of retention system for implant restorations [10], the primary purpose of this study is to investigate the frequency of implant restoration retention systems.

## 2. Materials and Methods

In April of 2017, a five-page questionnaire was sent to dental institutions, schools, and hospitals that provide dental services in different regions of Saudi Arabia. A total of 120 surveys were sent to 21 dental institutions. The questionnaire asked the respondents for general information, including their city, email address, institution/school, and their specialty. The survey included a total of eleven questions (Table 1).

All questions were of a multiple choice format and allowed the respondent to choose multiple answers. The questions were proofread and pilot-tested at the Faculty of Dentistry at Umm Al-Qura University by a prosthodontic staff member prior to distribution. The surveys were mailed to people in three groups: private institutes, educational institutes, and governmental hospitals. Data were analyzed with descriptive statistics using Microsoft Excel, version 15.19.1.

## 3. Results

**Figure 1:**
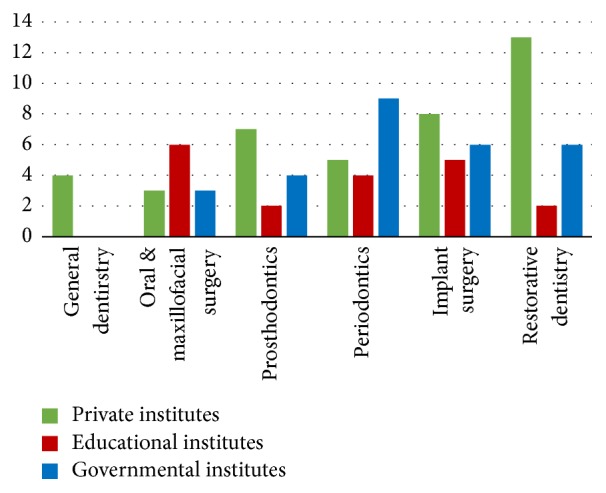
Clinician specialty of respondents.

In total, 120 surveys were distributed and 87 surveys were returned, for a response rate of 73%. This included thirty-six surveys (41.4%) from private institutes, twenty-two surveys (25.3%) from educational institutes, and twenty-nine surveys (33.3%) from governmental hospitals. Clinician specialty of the respondents included restorative dentistry (*n* = 21 (24.14%)), implant surgery (*n* = 19 (21.84%)), periodontics (*n* = 18 (20.69%)), prosthodontics (*n* = 13 (14.94%)), oral and maxillofacial surgery (*n* = 12 (13.79%)), and general dentistry (*n* = 4 (4.60%)) (Figure 1).

### 3.1. Private Institutes

**Figure 2:**
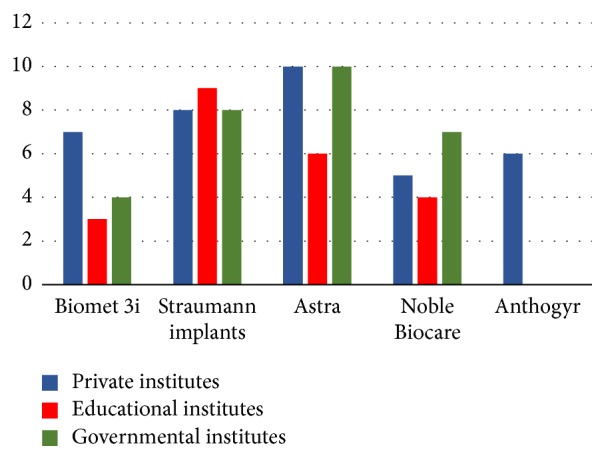
Implant manufacturers products: Biomet 3i, Inc. (Palm Beach Gardens, Florida); Astra Tech, Inc. (Waltham, Mass); Nobel Biocare AB (Göteborg, Sweden); Straumann USA, LLC (Andover, MA); and Anthogyr (Sallanches, France).

Private institute respondents revealed five commonly used implant manufacturers: Astra TECH implant system (*n* = 10 (28%)), Straumann dental implant system (*n* = 8 (22%)), Biomet 3i dental implant system (*n* = 7 (19%)), Anthogyr implants system (*n* = 6 (17%)), and Noble Biocare implant system (*n* = 5 (14%)) (implant manufacturer information is presented in Figure 2).

**Figure 3:**
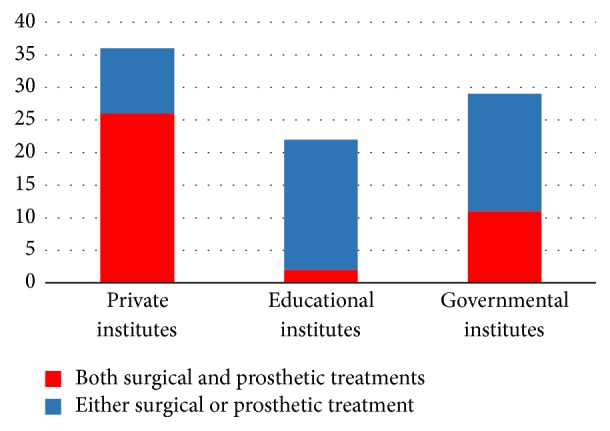
Role of the clinicians in a dental implant treatment.

Twenty-six (72%) respondents reported that the role of the clinicians in dental implant treatment was in both surgical and prosthetic treatments. Ten (28%) respondents reported that their role was limited to either surgical or prosthetic treatment (Figure 3).

**Figure 4:**
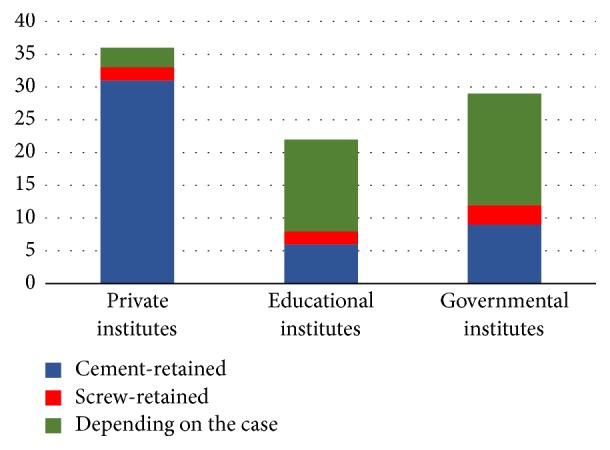
Type of retention system.

**Figure 5:**
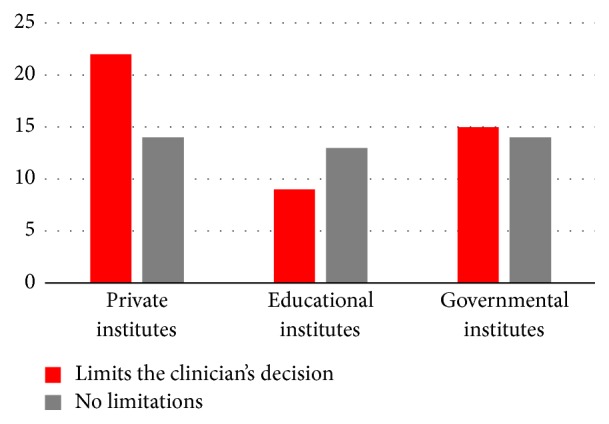
Role of the laboratory production.

Thirty-one (86%) respondents used cement-retained prosthetics in their practice more than screw-retained prosthetics, while 3 (8%) respondents reported that it depended on the case (Figure 4). Furthermore, laboratory production limits the clinician's decision in retention system type by 62% (*N* = 22); 38% (*N* = 14) reported no limitations (Figure 5).

For the access hole filling material, our study revealed that twenty-six (72%) respondents used the light cure composite resin, filled partially with cotton pellets; six (17%) respondents used the resin-modified glass ionomer, partially filled with cotton pellets, and four (11%) respondents used the light cure temporary filling, partially filled with cotton pellets.

**Figure 6:**
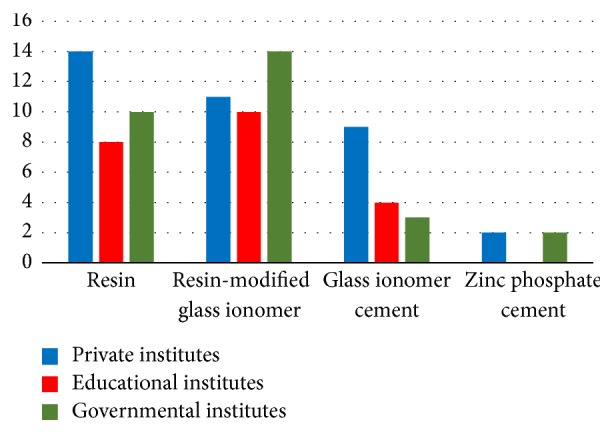
Definitive cementation material for the final implant restorations.

The most frequently used luting agent for the definitive cementation of the implant restorations was resin (*n* = 14 (39%)), followed by a resin-modified glass ionomer (*n* = 11 (31%)), glass ionomer cement (*n* = 9 (26%)), and zinc phosphate cement (*n* = 2 (1%)) (Figure 6).

**Figure 7:**
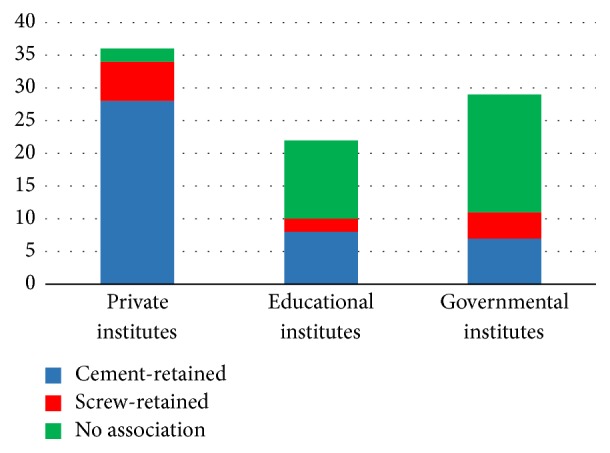
Failure of dental implants with an association with a retention system.

The last survey question asked about the failure rate of the dental implants, in association with the retention system. The results reveal that 28 (78%) respondents reported that the cement-retained restoration more frequently resulted in a dental implant failure, while 6 (17%) respondents reported that the failure was associated with the screw-retained system. Moreover, 2 (6%) respondents reported no association between the retention system and the implant failure (Figure 7).

### 3.2. Educational Institutes

Educational institute respondents revealed four commonly used implant manufacturers for dental school implants: Straumann dental implant system (*n* = 9 (41%)), Astra Tech implant system (*n* = 6 (27%)), Nobel Biocare implant system (*n* = 4 (18%)), and Biomet 3i dental implant system (*n* = 3 (14%)) (Figure 2).

Twenty (92%) respondents reported that the role of the clinicians in a dental implant treatment was limited to either a surgical or prosthetic treatment. Only two (8%) respondents reported their role in a dental treatment as both surgical and prosthetic (Figure 3).

Fourteen (64%) respondents reported that the retention system they used depended on the case. Six (27%) respondents preferred cement-retained rather than screw-retained prosthetics (Figure 4). Furthermore, the laboratory production limited the clinician's decision in the retention system by 41% (*n* = 9), while 59% (*n* = 13) reported no limitations (Figure 5). In relation to access hole filling material, our study reported that twelve (55%) respondents used the light cure composite resin, filled partially with cotton pellets, eight (36%) respondents used the resin-modified glass ionomer, partially filled with cotton pellets, and two (9%) respondents used the light cure temporary filling, partially filled with cotton pellets.

The most frequently used luting agent for the definitive cementation of implant restorations was the resin-modified glass ionomer (*n* = 10 (45%)), followed by resin (*n* = 8 (36%)) and glass ionomer cement (*n* = 4 (18%)) (Figure 6).

The last survey question asked about the failure of the dental implant in association with the retention system. 12 (55%) respondents reported that there was no association between the retention system and the dental implant failure, while 8 (36%) respondents reported that the failure was more frequently associated with cement-retained restorations. Only two (9%) respondents stated that it was more frequently associated with screw-retained restorations (Figure 7).

### 3.3. Governmental Hospitals

In total, 38% of the surveys were from governmental hospital workers that provided dental services. The results revealed four commonly used implant manufacturers: Astra Tech implant system (*n* = 10 (34%)), Straumann dental implant system (*n* = 8 (28%)), Nobel Biocare implant system (*n* = 7 (24%)), and Biomet 3i dental implant system (*n* = 4 (14%)) (Figure 2).

Eighteen (62%) respondents reported that the role of the clinicians in a dental implant treatment was limited to either surgical or prosthetic treatment. Eleven (38%) respondents reported their role in dental treatment as both surgical and prosthetic treatments (Figure 3).

Seventeen (59%) respondents reported that the use of the retention system depended on the case, while nine (31%) respondents preferred cement-retained systems to screw-retained systems (Figure 4). Furthermore, the laboratory production limits the clinician's decision in the retention system by 52% (*N* = 15); 48% (*N* = 14) reported no limitations (Figure 5). In terms of access hole filling material, our study reported that fifteen (52%) respondents used the light cure composite resin, filled partially with cotton pellets, nine (31%) respondents used the resin-modified glass ionomer, partially filled with cotton pellets, and five (17%) respondents used the light cure temporary filling, partially filled with cotton pellets.

The most frequently used luting agent for the definitive cementation of implant restorations was the resin-modified glass ionomer (*n* = 14 (48%)), followed by resin (*n* = 10 (34%)), glass ionomer cement (*n* = 3 (10%)), and zinc phosphate cement (*n* = 2 (7%)) (Figure 6).

The last survey question asked about the failure of the dental implant in association with the retention system. 18 (62%) respondents reported that there was no association between the retention system and the dental implant failure. 7 (24%) respondents reported that the failure was more frequently associated with cement-retained restorations. Only four (14%) respondents stated that is was more frequently associated with screw-retained restorations (Figure 7).

## 4. Discussion

The results of this study indicate that people working in a variety of specialty areas are involved in implant treatment. In addition, a wide range of implant manufacturer products and a wide range of implant retention protocols and cementation materials are used in their practice. This study also revealed commonly used implant manufacturers and techniques among dental clinicians in Saudi Arabia.

Tarica et al. [11] found that the most commonly used implant manufacturers in USA were Nobel Biocare, Biomet 3i, and Straumann. In Saudi Arabia, the most common implant systems were Astra, Straumann, Nobel Biocare, and Biomet 3i. Other implant companies include Dentium, Bego, Axiom, RePlant Implant, and BioHorizons.

Most dental clinicians in Saudi Arabia followed the American style in implant placement, while the role of the clinicians in implant treatment varied from one institute to another. In a private institute, the role of the clinicians was to perform both the surgical and prosthetic treatment. In an educational institute or governmental hospital, this was not the case. Perhaps this is due to restricted policies in educational and governmental hospitals in Saudi Arabia. In addition, it may be that a variety of specialties exist in governmental hospitals and educational institutes, which limits the clinicians to perform duties beyond their capabilities.

In a systematic review, a comparison was conducted between the cement-retained versus screw-retained restoration for marginal bone loss. Overall, the cement-retained restoration provided fewer prosthetic complications and a higher implant survival rate than screw-retained [12] restorations. In this study, the respondents were asked which retention protocols were used in their practice. The answer varied between the institutes. In general, cement-retained restorations were more frequently used than screw-retained restorations. The next survey question asked the respondents about the lab technicians' influence on the implant treatment. The results showed a variation among institutes. In private institutes, the lab technicians limited the retention systems selection. This may be due to the cement-retained restorations being relatively inexpensive to fabricate, requiring fewer laboratory skills and providing a better esthetic outcome [13]. On the other hand, the educational institute and governmental hospitals are totally funded by the government, which means that the cost of the fabrications is not present in the equation.

The sealing of the access hole of the screw-retained restorations is generally conducted with a partial filling with a cotton pellet and composite resin restoration. In a private institute, they rarely used the amalgam restoration to fill the hole. Most institutions used the resin-modified glass ionomer, resin cement, glass ionomer cements, and zinc phosphate cement. Their agreement in cement materials for definitive cementation indicates that the same cements are selected, due to convenience, familiarity, and cost. Some studies have shown that the cement used for natural dentation does not necessarily correlate with the cement used in dental implant restoration [14, 15].

The last survey question asked about the association between dental implant failure and retention systems. In general, respondents considered the cement-retained dental implant to be more often associated with dental implant failure. The educational institute respondents stated that the dental implant failure was not associated with the retention systems. In a systematic review conducted by Lemos et al. [12], the cement-retained implant resulted in less marginal bone loss when compared with screw-retained implants.

Screw loosening is a major problem with screw-retained restorations [16, 17]. The incidence of screw loosening was 65% for single tooth implant restoration [16], whereas the incidence of unretained cemented implant restoration was less than 5% [18]. However, it is possible to leave excess cement around the implant restoration, which leads to local inflammation and peri-implant disease, due to the microbiota populating the excess cement [19, 20].

However, this study also confirmed further investigation to expand the sample size. This may lead to more in-depth knowledge about the reasons behind implant failure.

## 5. Conclusions

Within the limitations of this study, the findings illustrate that Astra was cited as the most widely used implant system. In addition, cement-retained restorations were more frequently used than screw-retained restorations. Moreover, resin modified glass ionomer cement was most frequently used for definitive cementation. However, dental implant failures were more commonly associated with cement-retained restoration as compared to screw-retained restorations.

## Figures and Tables

**Table 1:** Survey questions.

(1) Please indicate your city.
(2) Please indicate your E-mail.
(3) Please indicate your institute.
(4) Please indicate your specialty.
(5) What implant system(s) is/are used in your practice?
(6) What is your role in implant treatment? (surgical part/prosthetic part)
(7) What retention systems do you use in your practice?
(8) Do the lab technicians limit your decisions in retention systems?
(9) What material(s) do you use to fill the access hole of the abutment screw?
(10) What cement(s) do you use for the final cementation of the implant restorations?
(11) From your practice, which retention systems are more frequently associated with failure?
